# Smart Helmet: Wearable Multichannel ECG and EEG

**DOI:** 10.1109/JTEHM.2016.2609927

**Published:** 2016-11-01

**Authors:** Wilhelm Von Rosenberg, Theerasak Chanwimalueang, Valentin Goverdovsky, David Looney, David Sharp, Danilo P. Mandic

**Affiliations:** Imperial College LondonLondonSW7 2AZU.K.

**Keywords:** Multichannel R-peak detection, QRS complex, wearable ECG, vital signs, wearable EEG

## Abstract

Modern wearable technologies have enabled continuous recording of vital signs, however, for activities such as cycling, motor-racing, or military engagement, a helmet with embedded sensors would provide maximum convenience and the opportunity to monitor simultaneously both the vital signs and the electroencephalogram (EEG). To this end, we investigate the feasibility of recording the electrocardiogram (ECG), respiration, and EEG from face-lead locations, by embedding multiple electrodes within a standard helmet. The electrode positions are at the lower jaw, mastoids, and forehead, while for validation purposes a respiration belt around the thorax and a reference ECG from the chest serve as ground truth to assess the performance. The within-helmet EEG is verified by exposing the subjects to periodic visual and auditory stimuli and screening the recordings for the steady-state evoked potentials in response to these stimuli. Cycling and walking are chosen as real-world activities to illustrate how to deal with the so-induced irregular motion artifacts, which contaminate the recordings. We also propose a multivariate R-peak detection algorithm suitable for such noisy environments. Recordings in real-world scenarios support a proof of concept of the feasibility of recording vital signs and EEG from the proposed smart helmet.

## Graphical abstract

We present a feasibility study of a smart helmet that uses embedded electrodes to record real-time electrocardiogram, respiration and EEG from face-lead locations. Recordings in real-world scenarios support a proof-of-concept of the feasibility of recording vital signs and EEG from the proposed smart helmet.

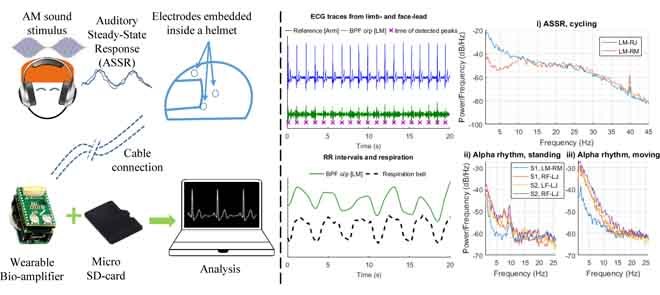

## I. Introduction

The monitoring of physiological signals using wearable devices is increasingly becoming a prerequisite for the assessment of the state of body and mind in natural environments. This has been facilitated by small-scale analogue and digital integrated circuit technology, together with on-chip processing power for dealing with movement induced artefacts in biopotentials, which are present when performing daily activities. Physiological signals recorded in real life tend to be notoriously weak and with a low signal-to-noise ratio (SNR). To this end, an amplifier with a high common mode rejection ratio is required; such high quality bio-amplifiers are typically integrated into the analogue front end of large stationary devices. Because of the many leads and electrodes required, such devices are well suited for clinical environments, where patients are normally stationary (except e.g. for cardiac stress tests), so that the noise level is relatively low.

### A. Motion Artefacts in Wearable Devices

Compared to stationary recordings, the measurements obtained from wearable devices are significantly more contaminated by noise because of subject movements. Real-world motion artefacts occur unpredictably and directly interfere with the signals of interest. Such artefacts are generated by (i) muscle contractions, recorded in an electromyogram (EMG); and (ii) an unstable contact between skin and electrodes. The latter causes imbalanced skin-contact impedances between bipolar electrodes which leads to a large difference in the biopotential at the analogue input [1]. These artefacts unavoidably affect the quality of the physiological signals because they produce an instantaneous, high amplitude, non-stationary disturbance which decreases the SNR of the acquired signals. In the frequency domain, the meaningful spectrum of the real signal can be buried under the dominating spectrum of the artefacts [2]. The frequency range of the artefacts introduced by electrode movements on the skin (0.01 Hz to 500 Hz) is similar to the surface EMG spectrum (2 Hz to 500 Hz) and both overlap with the significant parts of the frequency spectrum of the electrocardiogram (ECG) (0.05 Hz to 100 Hz), conventional electroencephalogram (EEG) (0.5 Hz to 100 Hz) [3], and full-band EEG (0.01 Hz to several hundred Hz) [4]. Applying standard filtering approaches to reject the artefacts will also remove a significant frequency band of the signal of interest. At the same time, while motion artefacts are a key problem in wearable devices, the power line noise is reduced compared to stationary devices. This is due to battery-powered amplifiers which typically use either SD-cards to store the data or wireless technology to receive and transmit data over a Gigahertz frequency range.

### B. The ECG and PPG

**FIGURE 1.**
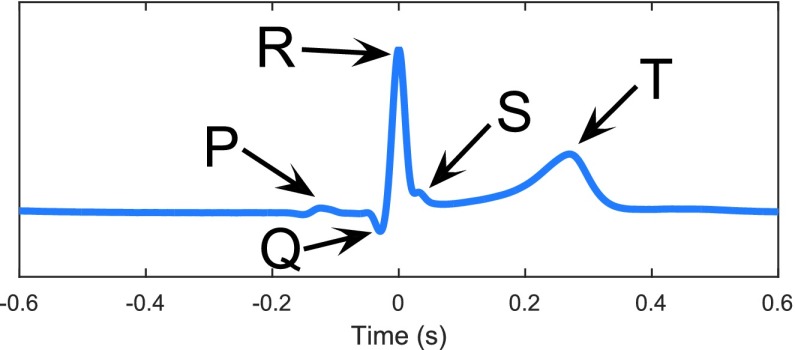
The ECG-cycle with its most significant features and labels.

The ECG has significant value both in clinical practice and outside the clinic, in terms of the ease of interpretation, reliability and physiological meaningfulness. In out-of-clinic applications, the activity of parasympathetic and sympathetic nervous systems can be estimated from the heart rate variability (HRV), a time series obtained from the time difference between consecutive R-peaks in the ECG, see Fig. 1. The relation between the power of the low frequency (LF) and high frequency (HF) bands of the HRV may indicate the degree of balance in the autonomous nervous system and thereby indirectly the level of physiological stress [5]. Apart from the ECG, the photoplethysmogram (PPG) is also widely used in wearable devices, most recently in ‘smart watches’. The PPG is based on reflections of a low power infrared light from pulsating blood vessels under the skin, e.g. from the wrist, earlobe or fingertip. However, the maxima in the recorded signal are wider than those in ECG their exact position (i.e. heart beats) cannot be identified with the same accuracy as QRS complexes in ECG; we therefore considered an ECG face-lead.

### C. Motivation

A variety of wearable ECG devices exists, however, most are used for measuring heart activity or calorie consumption in sports and can only compute an estimate of the heart rate. These are therefore not suitable for real-world activities where it is essential to record and monitor vital signs in uncertain or dangerous situations. One example are traffic accidents [6], especially when the state of body and mind of drivers, such as drowsiness, stress, anxiety and sickness, prevents them from concentrating on the road. A number of life-threatening injuries occur in cycling, motorcycle and car racing, horse riding, rugby, and cricket. This has motivated us to create a ‘smart helmet’ which can record and monitor both vital signs (ECG and respiration) and neural activity (EEG) of wearers.

A range of studies propose to measure vital signs from head locations, however, most focus on hardware development only, without taking into account signal processing techniques for the suppression of noise and artefacts in real-world signals. An example is found in [7] where in a Formula One racing car the relationship between the car speed and the heart rate of the driver was examined using wired limb-lead ECG. This comes with the disadvantage of recording equipment physically disturbing a driver, and a considerable setup time. To this end, unobtrusive wearable devices are being investigated [8], such as an army helmet which records the electrooculogram (EOG) and ECG of soldiers sitting at rest [9], whereby the sensors were positioned on a sweatband and a jaw strap. Although the motivation in the latter was to measure the level of consciousness, drowsiness and fatigue in soldiers, all recordings were performed at rest and using comparatively large recording devices. The use of a ballistocardiogram (BCG) and single-lead ECG to monitor the cardiac rhythm from behind the ear was studied in [10], where BCG-devices used accelerometers to measure the vibrations of the head produced by the pumping heart. The results show a correlation between the two measured signals. A further study investigated the recording of ECG from the ear [11], based on the potential difference between the right ear (using a modified earphone) and the left arm, in a configuration called ear-lead ECG. However, as ECG signals at the arm are much stronger than on the head, it can be assumed that the greater share of the potential changes originated on the left arm. In our own work [12], EEG was recorded inside the ear canal with a wearable device called ear-EEG (earEEG).

### D. R-PEAK Detection in ECG

The ECG recorded from wearable devices requires robust signal processing techniques for artefact removal. The crux of the analysis is to locate the R-peaks (see Fig. 1) in order to estimate the heart rate and compute the HRV. Among the many techniques for R-peak identification in noisy ECG, matched filtering has become a standard; this approach uses the QRS complex as a pattern and seeks to identify similar patterns [13]. This approach has been combined with neural networks for adaptive matched filtering [14] with the aim of automatically updating the QRS pattern that yields the highest accuracy, while in [15] matched filtering was performed in real-time for R-peak detection. The application of Hilbert transform is another common approach whereby the R-peaks are extracted from the envelope of ECG data [16], [17]. This approach is robust to baseline drift owing to a bandpass filter and a differentiation used in the pre-processing stage [18]. The approach by Pan and Tompkins (PT) [19] yields a high sensitivity of QRS complex detection in comparatively noise-free ECG and has become a standard for benchmarking purposes.

Our earlier study proposed an algorithm for detecting R-peaks in noisy ECG from wearable devices using a combination of matched filtering and Hilbert transform [20]. The approach is semi-automatic in the sense that the user can select a prototype QRS-waveform from raw ECG; the algorithm then runs automatically until an anomaly or a high noise level is identified; the user can make a decision on the location of the next peak. The algorithm is supported by a graphical user interface and was tested on the Physionet QT database and wearable ECG recordings from inside a helmet.

### E. Aims of the Proposed Study

We provide a proof-of-concept study of the feasibility for EEG and ECG recordings from within a helmet, and refer to this device as the smart helmet. In combination with the physiological responses derived from ECG and EEG, such as respiration via respiratory sinus arrhythmia (RSA), EMG via accelerometers, movement, and temperature, this promises a feasible tool for examining the state of body and mind of a user wearing the smart helmet. More precisely, the two main aims of this work are: (i) to introduce a smart helmet which can record the ECG and EEG without a decrease in comfort or any inconvenience to the user and in real-world scenarios, thereby exhibiting truly wearable characteristics; and (ii) to propose robust multivariate signal processing for the identification of R-peaks in noisy ECG and the detection of EEG responses. We are not aware of any results which measure EEG from inside a helmet or of R-peak detection algorithms that utilise several ECG-channels. This approach ensures a high accuracy even in noisy conditions.

## II. Background and Experimental Setup

### A. Electrocardiogram

The cells in the heart muscle are activated by electrical currents. As soon as one cell receives the impulse to contract, it transmits the message to its neighbouring cells and an area of cells starts to act effectively simultaneously. Delay lines with lower signal speeds between different areas of the heart ensure that the various parts operate in the intended order and that the four chambers contract in an organised way. A global model considers the superposition of all electric dipoles across the individual cell membranes to be caused by one electric dipole, the so called Heart Vector, for which the orientation and amplitude change cyclically over time [21]. Two electrodes attached to the body measure the projection of the Heart Vector onto the line that connects the two sensors so that the shape of the recorded ECG cycle varies according to the electrode positions. The peaks and minima in an ECG have assigned labels, with the R-peak being the most prominent feature (see Fig. 1). The knowledge of the timing of R-peaks allows for the determination of the heart rate via the inverse of the time difference between two consecutive R-peaks.

The accuracy of an algorithm for the identification of R-peaks is often measured by the parameters sensitivity (*Se*) and positive predictivity (+*P*) which account for the number of correctly and incorrectly identified and undetected R-peaks as follows [22]:

\begin{equation*} Se = \dfrac {TP}{TP + FN} +P = \dfrac {TP}{TP + FP} \end{equation*}

where the symbol *TP* denotes the number of accurately detected R-peaks, *FN* the number of missed R-peaks and *FP* the number of incorrect R-peak labels. The ground truth ECG is recorded from positions with a very high signal-to-noise ratio, such as on the arms or across the chest. An R-peak is considered as correctly determined, if the algorithm identified a peak within $[t_{R}-\Delta t, t_{R}+\Delta t]$ with $t_{R}$ as the correct time of the R-peak from the reference ECG and $\Delta t = 10$ ms.

For helmet-worn ECG sensors, the short distance between the sensing and reference electrodes on the head and the fact that the small neck area attenuates the electric potentials generated by currents in the heart make amplitudes of cardiac signals recorded on the face substantially smaller than those of ECG measured on the torso. Moreover, the movement of the head, eye blinking, jaw clenching, and swallowing cause complex motion artefacts in the recorded ECG, a subject to this study.

### B. Electroencephalogram

**FIGURE 2.**
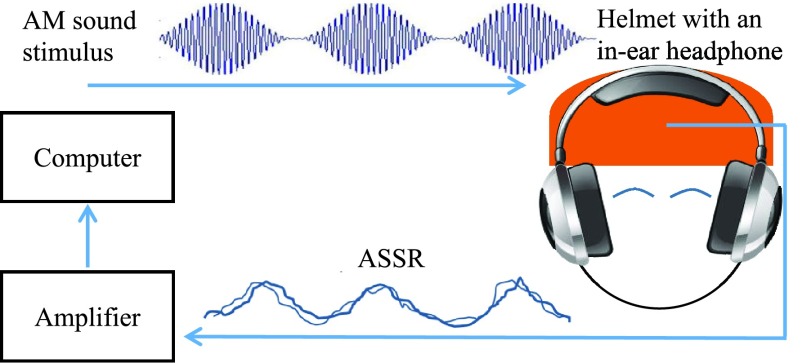
The experimental setup for recording ASSR: An amplitude modulated sinusoid is played to the subject via in-ear headphones inside a motorcycle helmet which records neural activity.

The ability of the proposed helmet to record electrical signals from the brain was assessed based on standard neural responses, such as the alpha rhythm in EEG (7.5 to 12.5 Hz) that is prominent when a person is in the state of wakeful relaxation with eyes closed. With an increase in the person’s activity the power of the alpha rhythm decreases. Additionally, two evoked response potentials (ERPs) were examined: (i) auditory steady state response (ASSR) and (ii) steady-state visual evoked potential (SSVEP). The ASSR is an auditory evoked potential, elicited in response to modulated tones played into the ear of the subject; the evoked EEG corresponds to the frequency of the envelope of the sound stimulus (see Fig. 2). A high-frequency sinusoid or white noise is amplitude modulated with a sinusoid of frequencies commonly around 19 Hz, 40 Hz or 80 Hz and played to the subject (in our study a 1 kHz sinusoid was amplitude modulated with a sinusoid of 40 Hz). This produced an EEG response corresponding to the modulating frequency in the temporal region of the brain (auditory cortex) and in the brainstem [23]. Note that the frequency spectrum of the amplitude modulated signal does not have a peak at the modulating frequency, and that the brain demodulates the input signal. The SSVEP response is similar to ASSR in that it is the response of the brain to external stimulation – in this case visual. The brain area which exhibits the strongest response is the occipital region, but the SSVEP can also be recorded from other regions, e.g. frontal and temporal. The standard stimulus is an LED blinking at a frequency between 3.5 Hz and 70 Hz, which results in an SSVEP response at exactly the same frequency [24].

## III. Experimental Configurations

### A. Sensor Positions

This section describes the four development stages (see Table 1) in our proof-of-concept study: from establishing that ECG can be measured from head locations using standard gold-cap electrodes, through to the feasibility of recording from user-friendly fabric electrodes in real-world scenarios.

**TABLE 1:** All Electrodes Were Passive Electrodes and the Type of Fabric was MedTex130. In Configuration 2, ECG and EEG Were Recorded, In the Other Three Configurations a Chest-Worn Respiration Belt Additionally Recorded a Reference Signal for the Respiratory Activity

#	Electrodes			Reference	Recorder	Condition
	Type	Size	No	method		
1	Gel	10 mm	11	Bipolar	Avatar	Stationary
2	Fabric	12 mm	7	Unipolar	g.USBamp	Stationary
3	Fabric	43 mm	7	Unipolar	iAmp	Moving
4	Fabric	12 mm	18	Bipolar	iAmp	Moving

**FIGURE 3.**
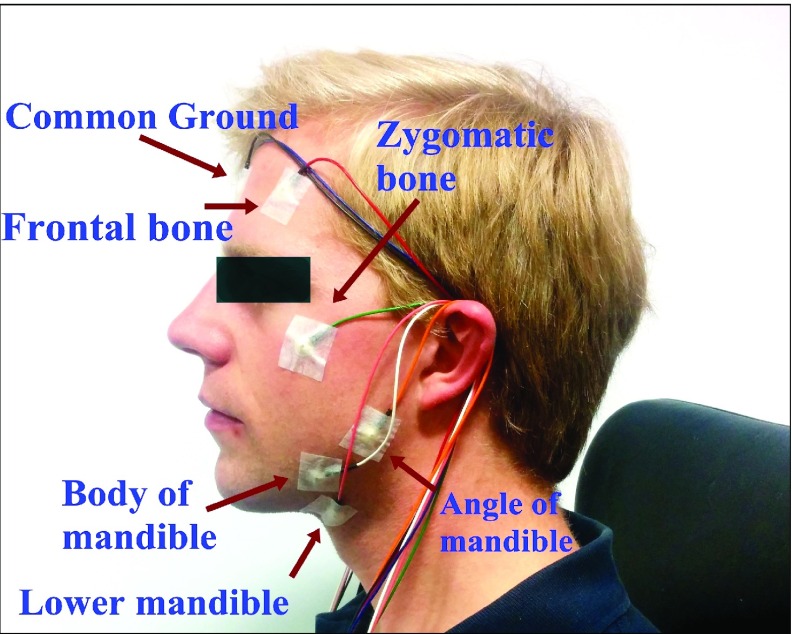
Recording Configuration 1: The electrodes were placed at ele- ven positions on the head and neck.

Based on the biophysics of ECG and EEG propagation and multiple trials, the optimal electrode placements for ECG and EEG recordings were suggested as follows. In Configuration 1, five bipolar measurements across the sagittal plane were set up. The ground (GND) was placed at the centre of the forehead while the positions on either side of the head were on the frontal bone, zygomatic bone, angle of mandible, body of mandible, and lower mandible, as illustrated in Fig. 3.

The initial configuration helped to identify suitable electrode positions which both gave satisfactory signal quality and were compatible with the helmet geometry. The user-friendliness was not a priority and therefore gold-cap electrodes filled with conductive gel for a low skin-electrode impedance were directly attached to the skin. Due to hair, the choice of possible locations for scalp EEG was limited, while there was more freedom in placing electrodes on the lower part of the head to record electric potentials from the heart. Out of the five described channels the lower three had the highest SNR for ECG (see Section VI-A).

**FIGURE 4.**
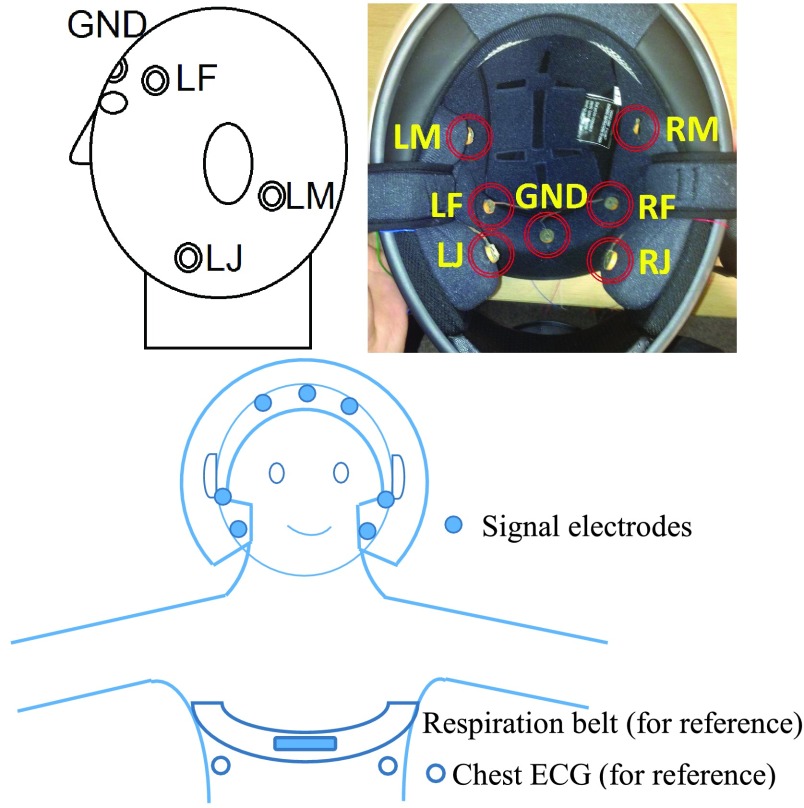
Upper: Configuration 2: Locations of the electrodes on the he- ad (left) and the setup inside the helmet (right). Lower: Location of the si- gnal and reference electrodes in all configurations.

In Configuration 2, the gold-cap electrodes were replaced by unobtrusive comfortable electrodes made from conductive fabric (MedTex130) with a diameter of circa 12 mm. Such electrodes can be sewn in to the inner lining of a helmet or replaced by capacitive electrodes [25]. Moreover, to be more flexible during the analysis and to enable re-referencing during the post-processing, the recording setup was switched from a bipolar to a unipolar setup. This also enabled re-referencing during the post-processing. Besides the GND electrodes at the identical position, six further electrodes were positioned at the **L**eft and **R**ight side of the face at the **J**aw, **M**astoid and **F**ore-head, as shown in Fig. 4.

For stationary recordings (see Section VI-B), Configuration 2 gave satisfactory signal quality, but exhibited a low SNR as soon as the subject started to engage in real-world activities such as walking or riding a bicycle. For a stable constant contact between skin and electrodes, the original small fabric electrodes were replaced by larger ones of circa 43 mm diameter in Configuration 3. However, the larger electrodes picked up more artefacts caused by slight movements of the helmet during physical activities (motion artefacts) and the SNR did not improve, compared to Configuration 2. In addition, the unipolar setup was susceptible to noise bursts in multiple channels simultaneously, which annihilated the advantage of a multichannel recording for identifying R-peaks. During the presence of motion artefacts in one channel, the remaining channels were also corrupted and the R-peak detection algorithm could not gain more information from another channel.

The proposed Configuration 4 employs two miniature eight-channel devices (see Section III-B) with an almost identical electrode setup. One channel of each device recorded the reference ECG on the chest which is used to synchronise the two devices (this step is not required when one device with more channels is utilised) and a further channel records the respiration reference, from a respiration belt. For the channels inside the helmet separate electrodes were used where possible even when a location was utilised multiple times; the recordings were bipolar. One data channel of each device measured the potential difference between the forehead and the left jaw and four further channels of each device recorded the potential differences around the jaw and the mastoid: LM-RJ, LM-RM, LJ-RJ, and LS-RS (S: on the **S**trap, but inside the helmet). Due to limited space on the inner lining, the LM electrode of the first device was used for two channels and the LJ electrodes acted as references for the forehead electrodes. This setup offers an advantage of almost completely independent channels with artefacts which are not directly related to one another, thus being amenable to multichannel R-peak identification.

The data channels between symmetric positions on each side of the head had a horizontal alignment and could therefore be compared to the standard Lead I configuration [26]. However, since the geometry of the body is irregular and the tissues between the signal source and the electrodes are inhomogeneous, components of the ECG from one or both of the remaining two Heart Vector orientations, vertical and horizontal (in the sagittal plane), can be picked up. For instance, this is the case for measurements between a jaw and a mastoid electrode on the same side of the head, whereby the maximum of the R-peaks can occur at a delay of a few milliseconds compared to the reference ECG on the chest [21].

**User comfort**: The subjects who took part in the studies based on the helmet with the fabric electrodes stated that the user experience was the same as when wearing a helmet without the sensors (with the exception of the reference ECG cables and the respiration belt which are not part of the actual smart helmet).

### B. Recording Devices

**FIGURE 5.**
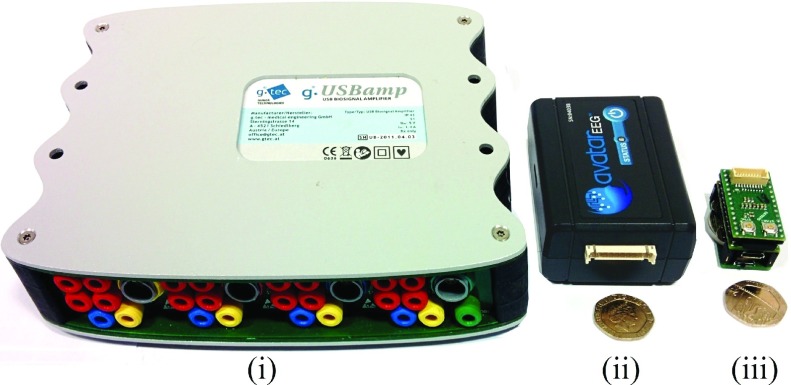
The three recording devices used: (i) g.USBamp by g.tec; (ii) Av- atar by EGI; and (iii) our own custom-made recorder with a 24 bit ADC. For reference, the devices are pictured against a 20 pence coin.

**FIGURE 6.**
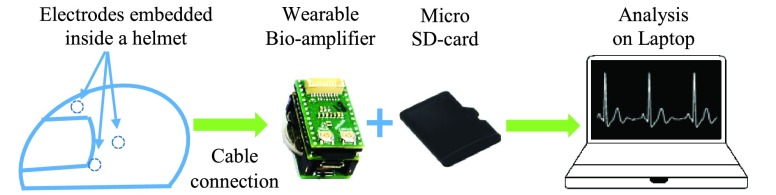
The final setup: Electrodes inside the helmet (and reference el- ectrodes) are attached to a biosignal-amplifier which writes data to an SD -card. The SD-card can be replaced with a Bluetooth adapter.

During our comprehensive testing stages, three different recording devices were employed, as shown in Fig. 5. The recordings in Configuration 1 were performed using the wearable Avatar recorder, manufactured by EGI, at a sampling frequency of fs = 500 Hz. Configuration 2 was only used as a proof-of-concept for a new electrode material and the stationary amplifier g.USBamp by g.tec was sufficient (fs = 1200 Hz). For Configurations 3 and 4, our own custom-made recorder (iAmp) based on the 24 bit analog-to-digital converter (ADC) ADS 1298 and the processor MK20DX256VLH7 was utilised (fs was set to 1000 Hz). Fig. 6 displays the whole setup, from the electrodes through to computer analysis.

## IV. Data Processing

The timing of R-peaks in the reference ECG was obtained using the software described in [20] and bandpass-filtered between 2 Hz and 70 Hz for the purpose of visualisation (3 $^{\mathrm{ rd}}$ order Butterworth filter). In the respiration channel, the significant frequencies are lower and a 3 $^{\mathrm{ rd}}$ order bandpass-filter with cut-off frequencies of 0.05 Hz and 15 Hz was used. For the neural responses the power spectral densities were produced using Welch’s method with window lengths of 4.8 s and window overlaps of 80%. Three 4 $^{\mathrm{ th}}$ order Butterworth filters were applied for which $\mathrm {f_{min}}$ and $\mathrm {f_{max}}$ were: (i) Alpha rhythm: $\mathrm {f_{min}} = 1$ Hz, $\mathrm {f_{max}} = 30$ Hz; (ii) ASSR: $\mathrm {f_{min}} = 1$ Hz, $\mathrm {f_{max}} = 45$ Hz; and (iii) SSVEP: $\mathrm {f_{min}} = 1$ Hz, $\mathrm {f_{max}} = 25$ Hz. The extraction of R-peaks from real world recordings involving movement was more complex and the approaches used are outlined in the next section.

## V. R-PEAK Identification

### A. Filtering Methods and Ranges

Configuration 1 (see Section III) was used to identify the most suitable electrode positions and to suggest the most robust signal processing tools. An initial result, based on one subject, was presented in [27] and five further persons were subsequently recorded. The three following filtering methods were investigated: (a) Bandpass filter (BPF); (b) Multivariate empirical mode decomposition (MEMD) [28]; and (c) Noise-assisted MEMD (NA-MEMD). For (b) and (c) 64 projection directions were taken and for (c) five channels with 20 dBm white Gaussian noise (WGN) were generated and the means over the individual intrinsic mode functions (IMFs) within NA-MEMD were calculated. The benefit of using MEMD and NA-MEMD is that they can effectively deal with and localise correlation in multichannel non-stationary data [29]. After applying these filtering techniques, the data were screened channel by channel and most information-bearing channels were used to compare different approaches.

The optimal frequency cut-offs for BPF and the best range of IMFs (all IMFs between the cut-off frequencies were summed) for MEMD and NA-MEMD were identified experimentally for all six subjects. The ‘frequency’ of an IMF was defined as its average instantaneous frequency, a method introduced in [30]. The investigated values for the lower and upper cut-off frequencies were $\mathrm {f_{min} \in [1, ~20] \in \mathbb {N}}$ and $\mathrm {f_{max} \in [max(f_{min}+5,6), ~40] \in \mathbb {N}}$. In all cases 3 $^{\mathrm{ rd}}$ order Butterworth filters were applied. Overall, 510 frequency ranges/IMF-compositions were analysed and the ideal cut-offs varied between subjects, depending on the individual properties of ECG-cycles, body geometries, and types of noise artefacts. Across all subjects, the frequency ranges of 9 Hz to 28 Hz for BPF, 7 Hz to 24 Hz for MEMD, and 10 Hz to 26 Hz for NA-MEMD yielded the highest ratios of accurately identified R-peaks.

**FIGURE 7.**
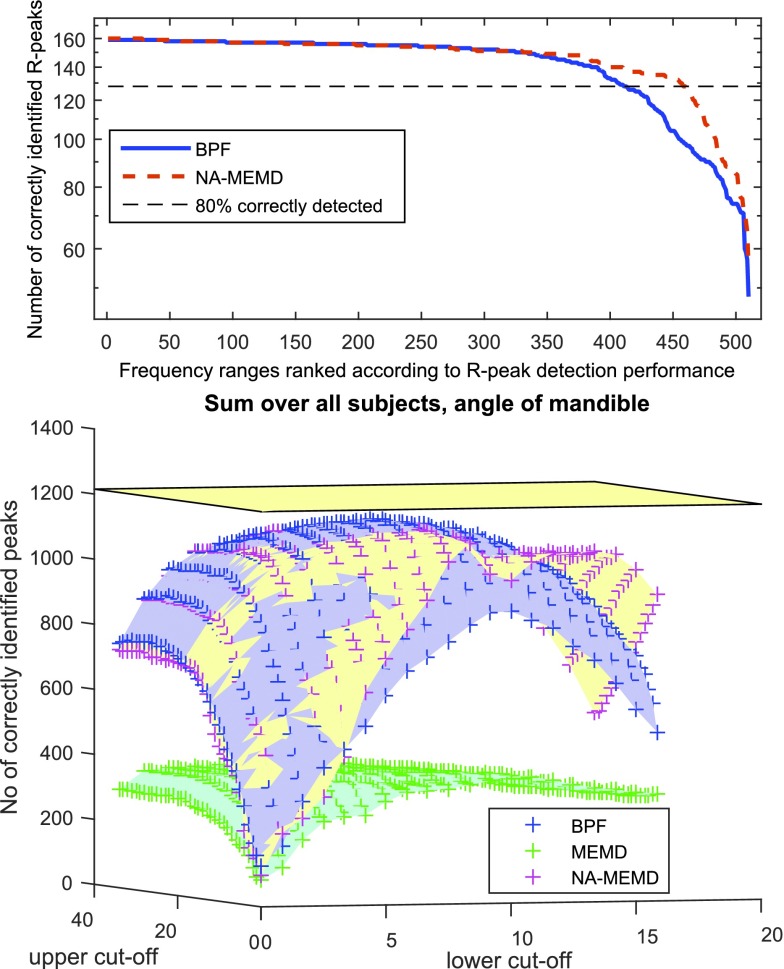
Performance of R-peak detection for different frequency ban- ds. Upper panel: NA-MEMD yields a wider range of satisfactory accuracy. Lower panel: Number of correctly identified R-peaks for every frequency range summed over all subjects at the AM electrode position (the yellow plane shows the total number of R-peaks ).

While BPF achieved the best results, an advantage of NA-MEMD is that for some subjects a larger number of frequency bins exhibits accurate R-peak detection results. Fig. 7 shows the fraction of correctly identified R-peaks against the frequency bins sorted according to performance quality. For NA-MEMD more frequency bins were able to detect a high fraction of R-peaks correctly, meaning that the cut-off frequencies can be chosen more freely. The 460 most accurate frequency bins for NA-MEMD identified at least 80% of R-peaks correctly while the performance was lower for BPF (413).

### B. Matched Filtering

Within the matched filtering approach, the measured ECG-trace is examined for a pattern QRS-complex, the part of the ECG-cycle between the Q- and the S-wave, see Fig. 1. One way to obtain a mother pattern would be to select a clear QRS-complex from the reference ECG, however, the ECG undergoes changes between the chest and the head and also depends on the electrode positions. Therefore, the values between 45 ms before and 45 ms after the R-peaks were taken from every channel individually. To attenuate noise, 20 ECG-cycles at rest were averaged whereby outliers were excluded. The exact timings of the R-peaks were obtained using a multivariate extension of the algorithm described in [27], the signals were bandpass-filtered and screened for peaks, followed by the calculation of the ratio of the amplitudes of the detected R-peaks and the overall average amplitude, for each channel. The channels with the eight highest ratios were chosen for the proposed multichannel R-peak detection. For each of the eight channels, the positions of identified R-peaks were marked in a matrix of the dimension $8\times \text{n}$ (n: length of the segment in sampling points; at the start all values are zero). In every row of the matrix, the timings of R-peaks in the respective channel were represented by isosceles trapeziums with a lower base of 21 samples, an upper base of 3 samples, and a height of 9 units. The most significant peaks in a row vector for which an element is the sum of the corresponding column in the matrix were labelled as R-peaks. This way, noise peaks of any amplitude which only occur in a few channels do not affect the detection algorithm, as long as a couple of channels point to the right location and the incorrectly detected peaks are not synchronised in time. The method to obtain the pattern QRS-complexes needs to be performed for relatively noise-free signals at the beginning of every session or alternatively once per subject.

The cross-correlations between the patterns and their recordings were next calculated and the R-peak search was performed on the correlation over time instead of applying the peak detection algorithm directly to the measured potential differences. The only filtering applied was in the pre-processing stage, where a high-pass filter with a cut-off frequency at 2 Hz was used to de-trend the signals which is essential for averaging and matched filtering.

### C. Multichannel R-PEAK Detection

Many artefacts present in physiological signals have an amplitude similar to that of R-peaks, together with sharing frequency components and waveforms. This all causes peak detection algorithms, time-frequency analysis, and matched-filtering to fail. However, owing to the fact that R-peaks occur in all data channels almost concurrently, the R-peak detection can be improved by taking multiple channels into account, as long as the artefacts only appear in relatively few channels simultaneously. The essential assumption is that when a peak appears at the same time in the majority of channels, it must be coming from a physiological source. There are very few limitations to this approach, for instance a strong mechanical impact to the helmet would temporarily disturb all electrodes.

In addition to the 10 actually recorded signals in Configuration 4, we also created 10 virtual channels by summing and multiplying the pairs of channels with neighbouring electrode positions. Again, a selection of the best seven channels was combined to identify the R-peaks, and the quality $Q$ of a channel was computed in the following way:

\begin{equation*} Q =\frac {\bar {R}-\bar {S}}{\overline {std(P_{QRS})}} \end{equation*}

where a horizontal bar denotes the mean, $R$ the amplitude of the pattern ±3 data samples around the occurrence of the R-peak, denoted $t_{R}$, $S$ the amplitude of the pattern ±3 data samples around ($t_{R} +30$ ms) and $std(P_{QRS})$ for the standard deviation of the individual 20 initial patterns from the previous section at every point in time.

The following steps were performed in the proposed multichannel R-peak detection:

1) Cross-correlate the recordings with the QRS-patterns;
2) Apply a peak detection algorithm to the N individual channels $\mathrm {i} = 1,2,...,N$, each of length L (L: length of the recording in sampling points) and save the peak locations in $\mathrm {lcs_{i}}$;
3) Create a zero-matrix $\mathrm {lcs_{c}}$ of the dimension $\mathrm {N} \times \mathrm {L}$;
4) In every row i of $\mathrm {lcs_{c}}$ the positions of identified peaks are marked with windows centred around $\mathrm {lcs_{i}}$. The window is the same isosceles trapezium as above and accounts for small time delays of peaks in different channels;
5) Sum $\mathrm {lcs_{c}}$ over the first dimension (over all channels);
6) Identify the peaks in the averaged signal from the previous step.

After Step 4), when plotting the rows of $\mathrm {lcs_{c}}$ over time, the specified window will be visible around every detected peak and for the rest of the time this value is zero, thus removing the impact of artefacts with high amplitudes. Averaging the recorded signals directly would emphasize those artefacts and corrupt potentially useful parts of the signal in other channels. In Step 6), time instances where peaks were simultaneously identified in multiple channels are prioritised without the risk of one channel dominating the others.

### D. R-Peak Prediction

A physically meaningful assumption that can be made for most subjects is that consecutive RR-intervals (RRIs) do not vary much around the local mean RRI. It should be mentioned that this limits the ability to detect ectopic heart beats.

Starting from a known R-peak, $\mathrm {R_{l}}$, (all trials started at rest) and an expected RRI, denoted by $\mathrm {RR_{e}}$ (the mean of the seven most recent RRI, at the beginning the mean RRI during the pattern-finding phase), the next $2\cdot \mathrm {RR_{e}}$ seconds of the measured signal after $\mathrm {R_{l}}$ were multiplied by a time window $TW$ with the length $2\cdot \mathrm {RR_{e}}$ the maximum of which coincides with $\mathrm {R_{l}}+\mathrm {RR_{e}}$. In this study, $TW$ was a Hanning window applied $w$ times, the weight $w$ could be adjusted to people or activities. In this way, potential peaks that occur around the time when the next R-peak is anticipated are given a higher priority over the peaks further away or closer to the previous peak. One disadvantage of this method is that when a series of spurious peaks was falsely selected as R-peaks, the expected position of the following R-peak can be shifted by fractions of a second, taking the algorithm longer to find its way back to the correct R-peaks and RRI. This effect is reduced when using multichannel R-peak detection described in Section V-C. Considering the structure of the multichannel detection algorithm, the R-peak prediction method is only superior when the individual channels suggest at least two different positions for the next R-peak.

### E. Multichannel R-PEAK Prediction and Detection

**FIGURE 8.**
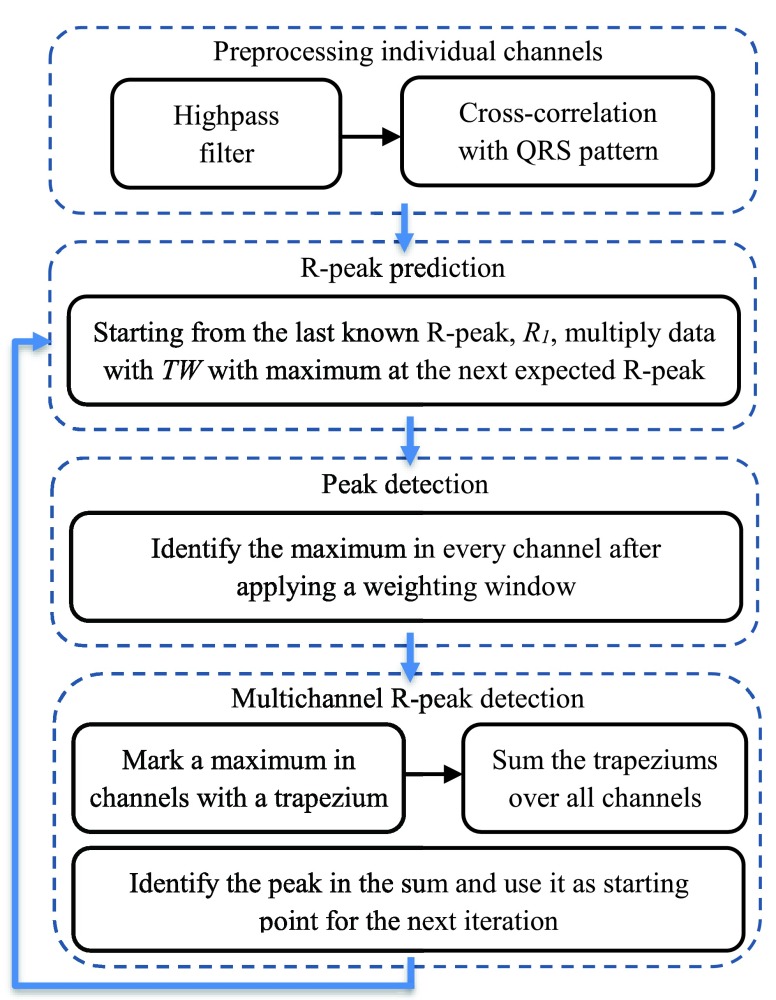
The steps of the combined R-peak prediction and multichannel R-peak detection algorithm.

The methods from the last two sections can be combined by firstly finding the cross-correlation between the QRS-patterns and the data channels, and secondly by applying the weighted time window $TW$ to the individual channels. The maximum value in each channel now becomes the location marker which is fed into the aforementioned multichannel algorithm. As shown in Fig. 8, after combining the candidate R-peaks, the height and area of the sum was taken into account when choosing the exact position of the next peak, and the so identified time instant becomes the starting point for the next iteration.

## VI. Experimental Results

The first set of experiments was used to establish the optimal electrode positions and suggest robust signal processing tools. For real-world conditions, Configurations 2 to 4 utilise the materials which were used in a study based on earEEG that included monitoring the stability of electrode impedance for 8 h [31]. Throughout the 8 h duration the impedance was stable and the same can be assumed for the helmet.

### A. Phase 1: Optimal Electrode Positions

Using Configuration 1 (see Section III), the ECG and EEG of six subjects were recorded for 170 s while seated at rest. As outlined in Section V-A, 510 different frequency ranges were analysed for five data channels and for six subjects. The final choice of electrode positions was made after the following steps: (1) finding the total numbers of correct and incorrect R-peaks over the subjects; (2) identifying the channels with the highest number of accurate R-peaks for each person and overall; (3) choosing the filter range with most correctly identified R-peaks; and (4) evaluating this frequency range for every individual (to check for person specificity). Table 2 displays the results, whereby the filter ranges which exhibited the best performance for the methods considered were: (i) BPF: 9 to 28 Hz; (ii) MEMD: 7 to 24 Hz; and (iii) NA-MEMD: 10 to 26 Hz. The MEMD only performed well for two subjects and the NA-MEMD performed almost as well as BPF. However, the computational effort for the former is much higher; for BPF, the overall Se and +P were 93.3% and 93% (excluding subject 4: 97.6% and 97.2%).

**TABLE 2:** Performance ($Se$ and+$P$ in %) of the Overall Best Frequency Band of Every Method in Total and for Each Subject at the AM Position in Configuration 1. The Symbol # Represents the Rank of the Frequency Band for Each Person. In 1020 Seconds, 1215 R-Peaks Occurred

Subj.	BPF			MEMD			NA-MEMD		
	Se	+P	#	Se	+P	#	Se	+P	#
All	93.3	93	1	30.3	32.5	1	92	91.8	1
1	99.5	99	2	97.4	96.9	4	96.4	96.4	4
2	98.7	98.3	2	2.1	2.6	7	96.1	95.7	5
3	96.6	96.6	2	1.5	1.5	7	96.6	96.1	3
4	75.1	74.8	1	3.9	4.6	4	73.4	73	1
5	98	97.5	2	6.1	6.2	2	97.4	97	2
6	94.4	93.8	6	94.4	93.8	1	95	95.6	5

**FIGURE 9.**
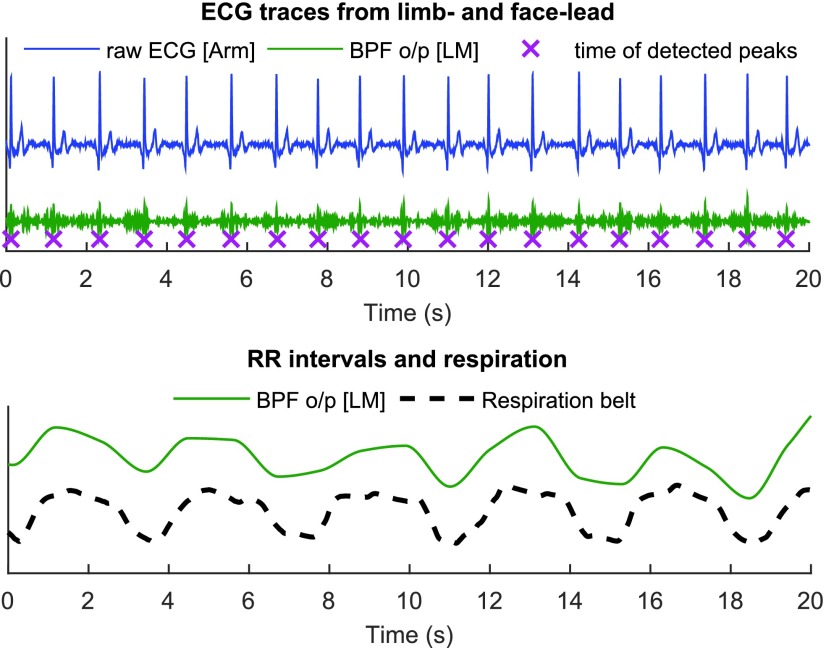
Recordings of ECG, RRI and Respiration. Upper: ECG recorded on left mastoid (LM) and its detected R-peaks compared to the reference from the arms. Lower: Respiratory activity influences the RRI, a phenome- non known as RSA. The dashed line shows the respiration recorded from a respiration belt and the solid line is the RRI time series obtained at LM.

Simultaneously, for reference, the respiratory activity was recorded with a chest-worn respiration belt. At rest, the RRI and respiration traces exhibited minima and maxima at about the same time instants, as shown in Fig. 9.

### B. Phase 2: Unipolar Setup with Soft Electrodes

In Configuration 2, based on previous results, the electrodes were moved towards the lower part of the head and were further attached at the mastoid positions. A unipolar setup enabled re-referencing of the measured potential differences. Preliminary results of this part were presented in [32] and the final ones are summarised in TABLE 3.

**TABLE 3:** Performance for Configuration 2 on Two Subjects Using Fabric Electrodes. HRD: Heart Rate Deviation - the Root-Mean-Square Error of the Difference Between the Estimated and the Real Heart Rate at Every Second

	Subject 1 (740 R-peaks)			Subject 2 (544 R-peaks)		
Setup	Se	+P	HRD (bpm)	Se	+P	HRD (bpm)
LJ-RJ	99.5%	99.1%	0.25	89.5%	88.9%	2.32
LM-RM	80.1%	78.9%	4.50	39.7%	39.2%	6.76
LM-LJ	99.6%	99.2%	0.33	99.4%	99.1%	0.21
LM-RJ	99.7%	99.5%	0.33	99.3%	98.9%	0.03
RM-RJ	98.6%	98.1%	1.32	65.8%	66.7%	4.41
LJ-RM	40.8%	40.5%	6.97	15.4%	14.6%	8.57
Fore-head	7.2%	7.1%	9.74	4.4%	4.1%	9.83

**FIGURE 10.**
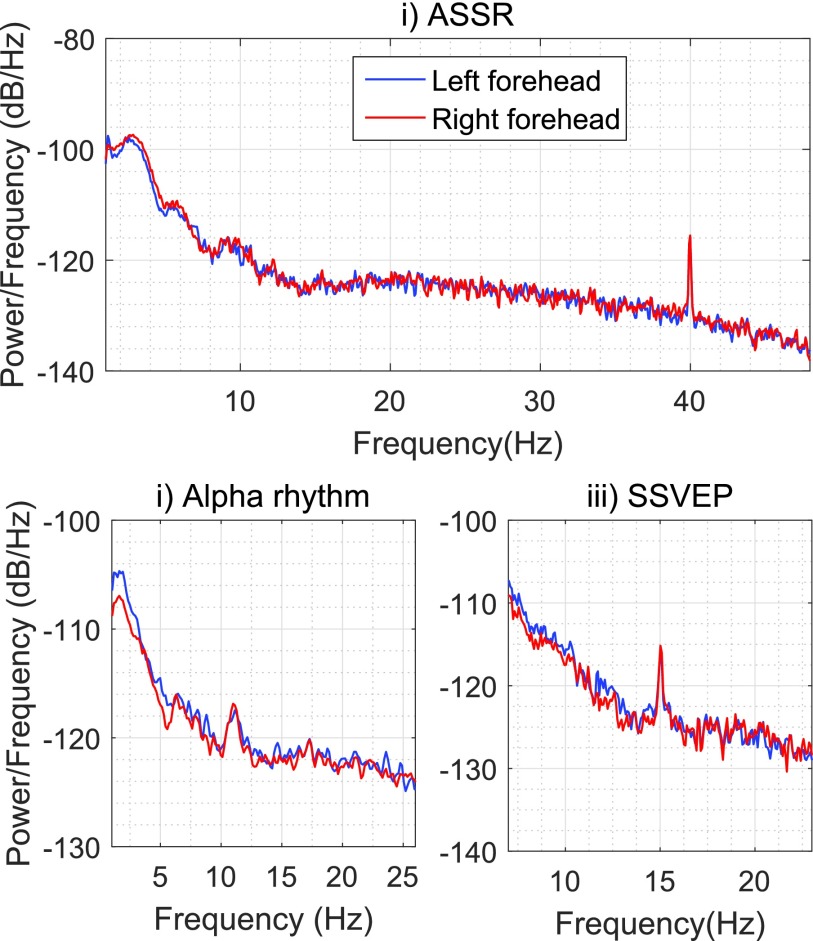
The PSD of three standard neural responses recorded from electrode positions amenable to a helmet: i) ASSR at 40 Hz; ii) Alpha rhy- thm (10 to 12 Hz); and iii) SSVEP at 15 Hz.

The recordings were performed under three conditions: (i) listening to a 1 kHz sinusoid amplitude-modulated with a 40 Hz sinusoid (ASSR); (ii) eyes closed (alpha rhythm which elicits an EEG response at ca 11 Hz); and (iii) watching an LED blinking at 15 Hz (SSVEP). Fig. 10 shows that all three responses could be recorded with both forehead electrodes, as indicated by the sharp peaks in the respective power spectra at the frequencies of 40 Hz, 12 Hz and 15 Hz.

### C. Phase 3: Performance in Real-World Scenarios

Using Configuration 2, the activities cycling on a road and walking yielded very high noise levels induced by the movement of the helmet, and thereby the movement of the electrodes against the skin (motion artefacts), which made it difficult to identify R-peaks in the recordings. To ensure a stable contact between the skin and the electrodes, the area of the electrodes was increased (Configuration 3, see Section III). The larger electrodes performed well when the subject was at rest but appeared to also enhance motion artefacts to the extent which made it impossible to reliably extract R-peaks (maximum *Se* and +*P*: 18.6% and 26.0%). A more effective approach was Configuration 4, where smaller electrodes picked up less noise while the rationale for using bipolar setups and two separate recording units was to avoid similar noise artefacts in multiple channels, which facilitates multichannel R-peak detection.

### D. Phase 4: Statistical Analyses in Real-World Scenarios

Two experiments were conducted to test the setup and algorithms under real-world conditions: one subject stood still for 60 s and then walked slowly for 60 s, while the second subject stood still for 67 s and then rode a bicycle for 43 s. TABLE 4 shows *Se* and *+P* during the activities, where for comparison the QRS-detection was also performed using the algorithm described in [19], referred to as the PT algorithm. For noisy data, the PT algorithm had a tendency to label too many peaks as R-peaks and therefore *Se* was high while *+P* was comparably low. Furthermore, for Subject 1 the difference in performance between the two best channels was large and therefore a channel with reasonably good results would need to be identified first, which is difficult. For Subject 2 all recording channels yielded unreliable R-peak detection when using the PT algorithm. In very few cases, after matched-filtering, the R-peak search in single ECG-channels led to similarly accurate results as the multichannel-R-peak search. However, the ‘good’ channels varied between people and trials, and therefore it is difficult to identify if and which single channels would enable precise R-peak detection. The advantage of the proposed method is that no prior knowledge about the quality of individual channels is required. Furthermore, the results can be improved by adjusting the weighting factor for the R-peak prediction to the subjects; for example *Se* and *+P* for Subject 2 could be increased to 98.6% during movement and to 99.4% overall. The computation time for the R-peak detection for Subject 2 with a trial length of 120 s was 2.5 s, out of which 0.7 s were needed to calculate the prototype QRS-patterns for all channels. The latter only needed to be performed once per subject.

**TABLE 4:** The$Se$ and +$P$ Indices During the Activities ’Standing’ and ’Moving’ Compared to the Best Two Channels when using a Standard R-Peak Detection Algorithm (PT 1 and PT 2) [19]

	Subject 1		Subject 2	
	Se	+P	Se	+P
Overall	96.9%	95.7%	96.7%	96.7%
Standing	98.8%	98.8%	100.0%	100.0%
Moving	94.9%	92.5%	91.5%	91.5%
PT 1	98.1%	77.6%	60.0%	48.0%
PT 2	73.6%	40.8%	57.8%	37.5%

**FIGURE 11.**
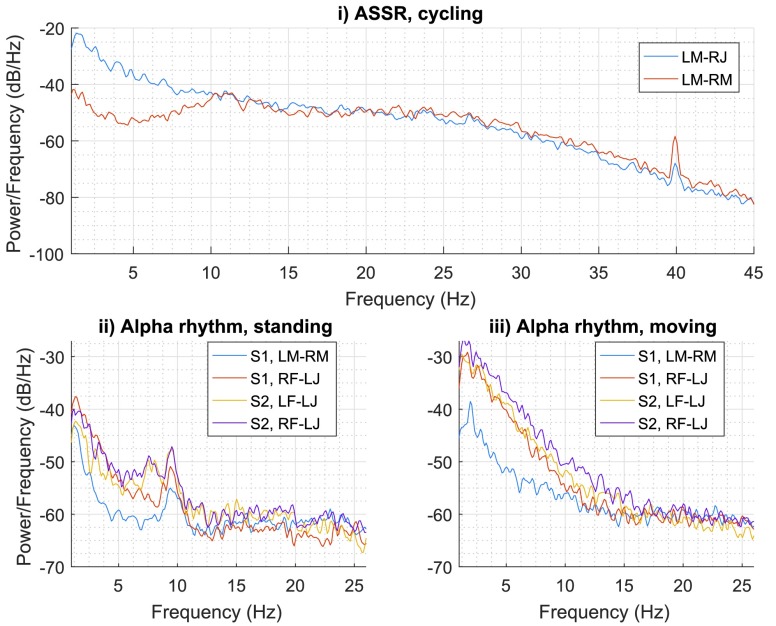
The PSD of the ASSR and alpha rhythm in EEG. i) ASSR of Subject 2 recorded for 43 s while cycling; ii) alpha rhythm of Subject 1 and 2 while standing; and iii) alpha rhythm of Subject 1 and Subject 2 while moving - Subject 1 : walking, Subject 2 : cycling.

At the beginning of the experiments, while standing, the subjects were asked to close their eyes and relax, in order to generate alpha waves in their EEG. Fig. 11 shows the EEG responses of the subjects, where the alpha rhythm – as expected (see Section II-B) – attenuated when the subjects opened their eyes and started the exercises, as observed when comparing the four lines in Fig. 11 ii) and iii). Moreover, the ASSR was induced by playing an amplitude modulated sound to the subject, as indicated by the peaks in the spectrum in both the blue and red lines. For safety reasons the SSVEP was not investigated while cycling or walking.

In this study, the same helmet was used for all subjects. However, the proposed technology can be applied to any helmet; since the helmets are chosen to fit tightly, this would reduce the effects of motion artefacts, thereby ensuring a good skin-electrode-contact. Additionally, the effect of a mechanical impact on the helmet, such as a speed bump, can be reduced using our newly developed Co-Located Multimodal Sensors [33], [34].

## VII. Conclusion

We have conducted a proof-of-concept study to demonstrate that electrodes mounted to the inside of a motorcycle helmet can reliably record cardiac and neural activity, together with respiration via a phenomenon called the respiratory sinus arrhythmia (RSA). The proposed recording setup has been shown to be very convenient, as it requires only the application of a saline solution to the soft electrodes embedded into the helmet lining. Recording of physiological signals has been conducted both at rest and while moving (walking and cycling). To deal with such noisy real-world scenarios, we have developed a signal processing approach based on matched-filtering and an adaptive weighting function for R-peak prediction across multiple channels. This has resulted in values for the sensitivity and positive predictivity parameters close to 100% at rest and over 90% during movement. The proposed recording of neural and cardiac activity from multiple locations has enabled accurate recordings even when some channels do not exhibit good skin-electrode-contacts. Another advantage of the proposed approach is that the developed signal processing algorithms do not require a priori knowledge of any parameters (for instance an approximate heart rate or a subject-specific threshold amplitude for R-waves), thus reinforcing the real-world nature of the proposed smart helmet recording.
